# Stress-Induced Reduction of Dorsal Striatal D2 Dopamine Receptors Prevents Retention of a Newly Acquired Adaptive Coping Strategy

**DOI:** 10.3389/fphar.2017.00621

**Published:** 2017-09-12

**Authors:** Paolo Campus, Sonia Canterini, Cristina Orsini, Maria Teresa Fiorenza, Stefano Puglisi-Allegra, Simona Cabib

**Affiliations:** ^1^Department of Psychology, Center ‘Daniel Bovet’, Sapienza Università di Roma Rome, Italy; ^2^Department of Psychiatry, University of Michigan, Ann Arbor MI, United States; ^3^Fondazione Santa Lucia (IRCCS) Rome, Italy

**Keywords:** dopamine receptors, dorsolateral striatum, helplessness behavior, hemispheric bias, memory consolidation, sustained threat

## Abstract

The inability to learn an adaptive coping strategy in a novel stressful condition leads to dysfunctional stress coping, a marker of mental disturbances. This study tested the involvement of dorsal striatal dopamine receptors in the dysfunctional coping with the Forced Swim test fostered by a previous experience of reduced food availability. Adult male mice were submitted to a temporary (12 days) reduction of food availability [food-restricted (FR)] or continuously free-fed (FF). Different groups of FF and FR mice were used to evaluate: (1) dorsal striatal mRNA levels of the two isoforms of the dopamine D2 receptor (D2S, D2L). (2) Forced Swim-induced c-fos expression in the dorsal striatum; (3) acquisition and 24 h retention of passive coping with Forced Swim. Additional groups of FF mice were tested for 24 h retention of passive coping acquired during a first experience with Forced Swim immediately followed by intra-striatal infusion of vehicle or two doses of the dopamine D2/D3 receptors antagonist sulpiride or the D1/D5 receptors antagonist SCH23390. Previous restricted feeding selectively reduced mRNA levels of both D2 isoforms and abolished Forced Swim-induced c-fos expression in the left Dorsolateral Striatum and selectively prevented 24 h retention of the coping strategy acquired in a first experience of Forced Swim. Finally, temporary blockade of left Dorsolateral Striatum D2/D3 receptors immediately following the first Forced Swim experience selectively reproduced the behavioral effect of restricted feeding in FF mice. In conclusion, the present results demonstrate that mice previously exposed to a temporary reduction of food availability show low striatal D2 receptors, a known marker of addiction-associated aberrant neuroplasticity, as well as liability to relapse into maladaptive stress coping strategies. Moreover, they offer strong support to a causal relationship between reduction of D2 receptors in the left Dorsolateral Striatum and impaired consolidation of newly acquired adaptive coping.

## Introduction

The ability to respond to stressors with adaptive coping strategies is determinant for the psychological well-being of human and non-human animals (Koolhaas et al., 2010; Maier and Watkins, 2010; Cabib and Puglisi-Allegra, 2012; Helmreich et al., 2012; Pomerantz et al., 2012; Andolina et al., 2013; de Kloet and Molendijk, 2016). Indeed, dysfunctional stress coping characterizes different mental diseases (Taylor and Stanton, 2007; Aldao and Nolen-Hoeksema, 2010; Moritz et al., 2016). Therefore, animal models of stress coping have major translational value for research.

Although immobility expressed by rodents in the Forced Swim test (FSt) has been used as a measure of depressive-like behavior, there is a large consensus on the view that this behavioral response is an adaptive strategy to cope with a stressful situation that cannot be avoided nor escaped (Cabib et al., 2012; Andolina et al., 2013; Campus et al., 2015; de Kloet and Molendijk, 2016). During their first FSt experience (10 min for mice 15 min for rats) animals show initial expression of vigorous active coping (swimming around and struggling to climb the container’s walls). These responses decrease overtime whereas episodes of immobility (only small movements required to keep the head above water) increase in frequency and duration. The immobility response prevents useless and risky loss of energy, thus it is acquired and consolidated as long-term memory to be immediately adopted on subsequent encounters with the stressor (Mitchell and Meaney, 1991; Colelli et al., 2014; Reul, 2014).

Development or expression of immobility in FSt is disrupted by proximal stress experiences (Molina et al., 1994; Alcaro et al., 2002; Becker et al., 2008; Mozhui et al., 2010), an observation that further support the translational value of this animal model because proximal adverse experiences contribute to development of mental diseases (Dias-Ferreira et al., 2009; Daskalakis et al., 2013; Gourley et al., 2013; Diwadkar et al., 2014; Reul, 2014; Eagle et al., 2015). The neurobiological mechanisms mediating the disruptive effects of proximal stress experiences on subsequent coping with FSt are still poorly understood, although disturbances of learning processes could play a major role (Mitchell and Meaney, 1991; Colelli et al., 2014; Reul, 2014).

In the present study we tested the involvement of dorsal striatal D2 dopamine receptors (D2R) in impaired consolidation of newly acquired passive coping response to FSt fostered by a temporary reduction of food availability. Indeed, strong evidence supports a role for the dorsolateral striatum in consolidation of a long-term memory of immobility in FSt (Colelli et al., 2014; Campus et al., 2015) and recent findings offer support to the involvement of impaired memory consolidation in stress-induced disruption of FSt coping in mice (Campus et al., 2016). Moreover, different types of stressors have been shown to alter availability of D2R in the mouse brain (Puglisi-Allegra et al., 1991; Cabib et al., 1998; Patrono et al., 2015) and D2R play a major role in memory consolidation (Sigala et al., 1997; Setlow and McGaugh, 1999; Manago et al., 2009; Lee and Chirwa, 2015). Finally, unseasonal reduction of food availability represents an unpredictable stressor in natural settings (Wingfield and Kitaysky, 2002), thus it models an ecologically meaningful stressor in laboratory settings.

## Materials and Methods

### Animals and Housing

Male mice of the inbred DBA/2J strain (Charles River, Como, Italy) were purchased at 6 weeks of age and housed in groups of four in standard breeding cages with food and water *ad libitum* on a 12-h dark/light cycle (lights on between 07:00 and 19:00 h) at a temperature of 22 ± 1°C.

At 7 weeks of age mice were all individually housed and assigned to free-feeding (FF) or food-restricted (FR) condition. FF mice received food once daily in a quantity adjusted to exceed daily consumption (15 g). FR mice received food once daily in a quantity adjusted to lose 15% of the initial body weight within the first 3 days and maintain the reached weight for the following 9 days. On the evening of the 12th day of individual housing mice from both FF and FR groups were given food *ad libitum* and left undisturbed for 48 h before behavioral testing or tissue collection.

All animals were treated humanely in accordance with the principles expressed in the Declaration of Helsinki. Experimental protocols and related procedures were approved by Italian Ministry of Public Health. All efforts were made to minimize animal suffering, according to European Directive 2010/63/EU on the use of animals for research.

### RNA Isolation and Gene Expression Analysis by Quantitative Real-time RT-PCR

**FIGURE 1:**
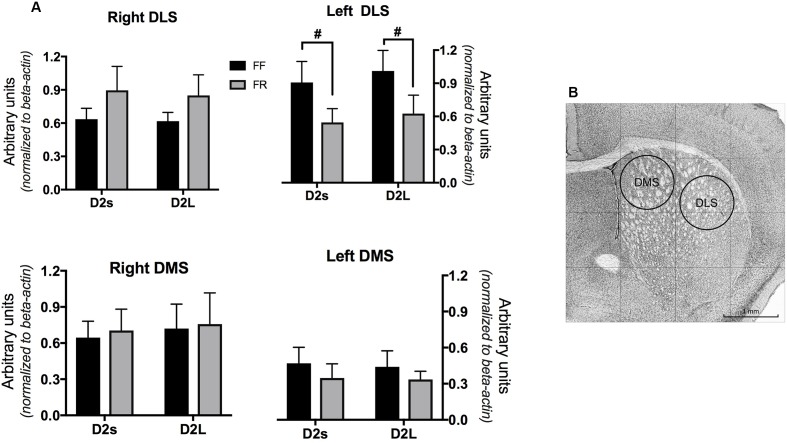
**(A)** mRNA level of the two isoforms of D2 dopamine receptors (short D2S; long D2L) in the dorsomedial and dorsolateral striatum of continuously free fed (FF) and temporarily food restricted (FF) mice. Data are expressed as mean arbitrary units ± SEM. **(B)** Punches location and dimension (example from the left hemisphere). ^#^Main effect of the feeding condition (see Results).

Mice were killed by cervical dislocation and then decapitated for brains excision. Brain was quickly removed, frozen in dry ice and sliced with a freezing microtome. A stainless steel needle (1 mm inside diameter) was used to punch DLS and DMS from coronal slices no thicker than 300 μm (three slices from 1.05 to 0.15 from bregma, MBL Mouse Brain Atlas DBA/2^1^). Locations of punches are shown in **Figure 1**.

Punches were processed for total RNA extraction using the RNeasy Mini kit (Qiagen). To reduce variability in the total RNA yields, the automated QiaCube instrument (Qiagen, Hilden, Germany) was used to purify RNAs. Purified RNA samples were treated with DNase I to remove genomic DNA and cDNA was synthesized using Superscript III reverse transcriptase (Life Technologies, Rockville, MD, United States), acc.to the protocol supplied by the manufacturer.

Real-time RT-PCR was performed on a EcoTM Illumina thermal cycle using QuantiFast Sybr Green PCR Master Mix (Qiagen, Inc., Valencia, CA, United States). Amplification reaction conditions were as follows: 95°C for 10 min, then 40 cycles of 95°C for 15 s and 58°C for 60 s.

Primer sequences were: D2L_forward: 5′-AACTGTACCCACCCTGAGGA-3′; D2S_forward 5′-CACCACTCAAGGATGCTGCCCG-3′; D2_reverse: 5′-GTTGCTATGTAGACCGTG-3′; β′actin_forward 5′-GAAATCGTGCGTGACATCAAAG-3′; β′actin_reverse 5′-TGTAGTTTCATGGATGCCACAG-3′. The size of amplified PCR product was 228 bp (D2L), 155 bp (D2S), and 216 bp (beta-actin).

The amplification efficiency for each primer pair was preliminarily determined by amplifying serial dilution (0.32–200 ng) of total striatum cDNA obtaining a linear standard curve. All primer pairs showed good linearity and similar amplification efficiency (98–99%) with all primers pairs. To control for products specificity, a melting temperature dissociation curve analysis was performed, from 55 to 95°C, measuring fluorescence every 0.5°C and the amplified PCR products were visualized after electrophoresis on 1.8% agarose gel. Each experiment was performed in triplicate and values were averaged. For each primer pairs, negative controls without cDNA were included to rule out genomic DNA contamination. Beta actin expression was found to be unaffected by our experimental design in all brain regions examined, and was therefore used as the internal control for normalization. Similar results were obtained when GAPDH was used for normalization.

### Forced Swimming Test

Apparatus and procedures were previously described (Colelli et al., 2014; Campus et al., 2015). Briefly, the procedure consisted of a first and a second experience separated by 24 h time interval. Each mouse was gently laid in the water contained in a glass cylinder and left for 10 min on the first experience (training) and 5 min on the second (test). Both sessions were registered by a digital video camera located frontally to the apparatus and connected to a computer located within a different room.

Duration (sec) of struggling to climb out, swimming, and immobility (absence of all movements not required to float), was scored on videotapes by a trained experimenter, unaware of the experimental groups each animal belonged to, using EthoVision (Noldus Netherlands).

### Immunohistochemistry

Mice were killed by cervical dislocation and then decapitated for brains excision. Brains were removed, post-fixed, and cryoprotected as described previously (Conversi et al., 2006; Colelli et al., 2010). Tissue was sliced on the coronal plane into 40 μm thick sections. Rabbit anti- c-fos (1/20000; Oncogene Sciences) was used as primary antibody. Peroxidase staining was obtained by standard avidin–biotin procedure using the rabbit Vectastain Elite ABC Kit (Vector Laboratories, Inc., Burlingame, CA, United States) and chromogenic reaction was developed by incubating sections with metal-enhanced DAB (Vector Laboratories, Inc., Burlingame, CA, United States), according to the protocol supplied by the manufacturer. Images of dorsolateral (DLS) and dorsomedial (DMS) striatum were acquired with a Nikon Eclipse 80i microscope equipped with a Nikon DS-5M CCD camera. The analysis of images was performed by using the public domain image analysis software IMAGEJ 1.48 (Abramoff et al., 2004). Immunoreactive nuclei quantification was expressed as density (number of nuclei/0.1 mm^2^).

### Temporary Inactivation of Dopamine Receptors in the Dorsolateral Striatum

After 1 week of individual housing, continuously free-fed (FF) mice were implanted with stainless-steel guide cannulas unilaterally in the left dorsolateral striatum (DLS) as previously described (Colelli et al., 2014; Campus et al., 2015). Briefly, mice were anesthetized with Zoletil 100, Virbac, Milano, Italy (tiletamine HCl 50 mg/ml+zolazepam HCl 50 mg/ml) and Rompun 20, Bayer S.p.A Milano, Italy (xylazine 20 mg/ml) purchased commercially, and mounted on a stereotaxic apparatus (David Kopf Instruments, Tujunga, CA, United States). A single stainless-steel guide cannula (Unimed, Geneva, Switzerland: 7 mm in length, 0.50 mm in external diameter) was inserted in the left DLS (+0.8 mm anterior to bregma, ± 2.3 mm lateral to midline, -2.5 mm ventral from the skull according to Franklin and Paxinos, 2001). One week later, drugs were infused (total volume 0.4 μl, flow rate of 0.2 μl/min) through a stainless-steel injection cannula (Unimed, Geneva, Switzerland, 0.11 mm in internal diameter) connected by polyethylene catheter tubing to a 1 μl Hamilton micro-syringes (Hamilton, Co., Reno, NV, United States), as previously described (Colelli et al., 2014; Campus et al., 2015). Infusions were performed immediately after the firs FSt experience and behavioral test was performed 24 h later.

Doses of dopamine agonists were chosen in preliminary experiments. The D1 dopamine receptor antagonist SCH 23390 (Sigma Aldrich) was dissolved in a 0.90% saline solution and infused at one of two doses (0.5 or 1 μg/mouse); the D2/D3 dopamine receptor antagonist sulpiride (Sigma Aldrich) was first dissolved in 100% dimethyl sulfoxide (DMSO) and then diluted in 0.90% saline up to reaching two different final concentrations (0.5 or 1 μg/mouse). The final DMSO concentration never exceeded 10%; 0.90% saline or 0.90% saline +10% DMSO were infused in control (0) groups.

### Experimental Protocols and Statistics

Because of previous evidences for the selective involvement of the left DLS in consolidation of a long-term memory of passive coping acquired in FSt (Colelli et al., 2014; Campus et al., 2015), we separately evaluated D2R mRNA levels and c-fos immunostaining in the left and right hemispheres and statistically tested a possible bias by including the factor ‘Hemisphere’ in the ANOVAs.

All FR mice were behaviorally tested or sacrificed after 12 days of restricted feeding followed by 48 h of free food availability (14 days of individual housing). All FF mice were behaviorally tested or sacrificed following 14 days of individual housing.

A total of eight groups of FR and of 14 groups of FF mice were used for the present experiment.

One group of FF and one group of FR mice (*n* = 6 each) were used to quantify D2R mRNA levels. D2R mRNA determinations obtained from triplicate experiments were averaged for each subject and statistically analyzed by three-way ANOVAs (Isoform = two levels: D2L, D2S; Feeding = two levels: FF, FR and Hemisphere = two levels: Left, Right). Additionally, we evaluated a possible difference in hemispheric availability of the two D2R isoforms in FF and FR mice separately, by two-way ANOVAs (Isoform × Hemisphere).

Two groups of FF and two groups of FR mice were used for c-fos immunostaining experiments. Six mice from each feeding condition were sacrificed immediately after removal from their home cages (naïve). Six mice from each feeding condition were sacrificed 50 min after a first experience of forced swim (10 min, FSt). Statistical analyses of c-fos data were performed on number of immunostained nuclei/0.1 mm^2^ from each sampled area. Three-way ANOVAs were used (two between factors: Experience of forced swim = two levels: Naïve, Experienced; Feeding = two levels: FF, FR; and one within factor: Hemisphere = two levels: Left, Right). When allowed by the results, individual between-groups comparisons were performed *post hoc* (Sidak correction).

A group of FF (*n* = 6) and a group of FR (*n* = 6) mice were used to evaluate the effects of a previous experience of restricted feeding on behavioral responses to FSt. Statistical analyses were performed on duration (sec) of the different behaviors expressed in the first and second block of 5 min of the first FSt experience and on the 5 min test performed 24 h later. A two-way ANOVAs for repeated measures (min blocks, three levels: 0–5, 6–10 min, test) with Feeding as between factor was employed. When allowed by results obtained, significant difference with behavior expressed during the first 5 min of exposure to FSt was tested *post hoc* (Paired *t*-test).

Six groups (one group for vehicle and one for each of the two doses of each antagonist) of FF mice (*n* = 7 each) were used to evaluate the effects of immediate post-experience infusion of DA antagonist in the left DLS on consolidation of a passive coping response (immobility). All mice were exposed to 10 min of forced swimming on the 14th day of individual housing and tested the following day. Immobility duration (sec) expressed on the final test was statistically analyzed by a two-way ANOVA for independent measures (Feeding, two levels = FF, FR; Dose, three levels = 0, 0.5, 1 μg/mouse). When allowed, individual between-groups comparisons were performed *post hoc* (Sidak correction).

## Results

### Restricted Feeding Selectively Reduced D2R mRNA Level in the Left Dorsolateral Striatum

**Figure 1** shows data on dorsal striatal D2L and D2S mRNA levels indicating a selective decrease of both D2R isoform limitedly to the left dorsolateral striatum (DLS) of FR mice.

Indeed, statistical analyses only revealed a significant interaction between the three factors (Feeding × Hemisphere × Isoform) in the DLS [*F*(1,40) = 7.307; *p* < 0.05] due to significant [*F*(1,10) = 7.29; *p* < 0.05] reduction of mRNA levels of both isoforms in the FR group (**Figure 1**).

Because larger D2R availability has been observed in the left striatum of rats and healthy humans (Schneider et al., 1982; Tomer et al., 2008), we separately compared hemispheric levels of the two D2R isoforms in the different striatal compartment of FF and FR mice. A significant hemispheric difference was found in the DLS of FF mice only, due to a left bias for both isoforms [*F*(1,20) = 5.136; *p* < 0.05].

### Restricted Feeding Selectively Prevented Forced Swim-Induced c-fos Expression in the Left Dorsolateral Striatum

**FIGURE 2:**
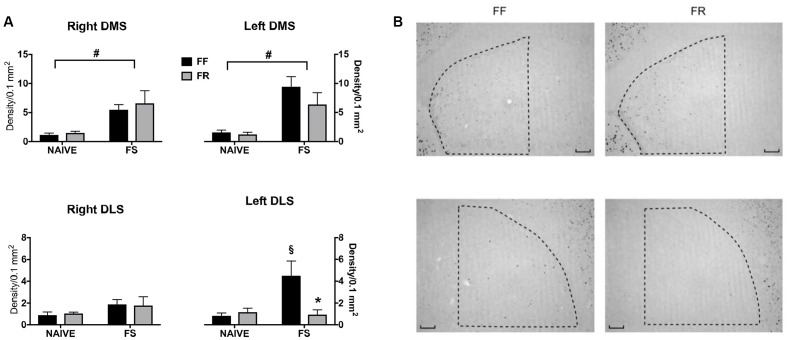
Effects of 12 days or restricted feeding on c-fos immunostaining promoted by a first experience (10 min) of Forced Swim. **(A)** Quantitative data (mean density/0.1 mm^2^ ± SEM). ^#^Main effect of the Forced Swimming experience (see Results). ^x^*p* < 0.005 vs. Naive (not exposed to FS) (Sidak correction for multiple comparisons). ^∗^*p* < 0.05 vs. continuously free-fed (FF) mice exposed to Forced Swim (Sidak correction for multiple comparisons). **(B)** Representative staining of samples from the left DMS (top) and left DLS of continuously free-fed (FF) and temporarily food-restricted (FR) mice exposed to 10 min of forced swimming.

In **Figure 2** are shown data on induction of c-fos immunostaining by a first FSt (10 min) experience in the right and left striatum of FF and FR mice. These data indicate a selective induction of c-fos expression in the left DLS of FF mice that was absent in FR mice.

Thus, statistical analyses revealed a significant main effect of FSt experience in the dorsomedial striatum (DMS) [*F*(1,40) = 38.36; *p* < 0.0001] due to a significant increase of c-fos immunostaining in FSt-experienced mice regardless of the hemisphere or of the feeding condition. A significant global interaction was revealed for c-fos expression in the DLS [*F*(1,40) = 4.182; *p* < 0.05] due to a significant c-fos expression in the left DLS of FF mice only (**Figure 2**).

### Either Restricted Feeding or Pharmacological Inactivation of D2/D3 Receptors in the Left Dorsolateral Striatum Prevented 24 h Retention of the Passive Coping Strategy Acquired in the First Experience with Forced Swim Test

**FIGURE 3:**
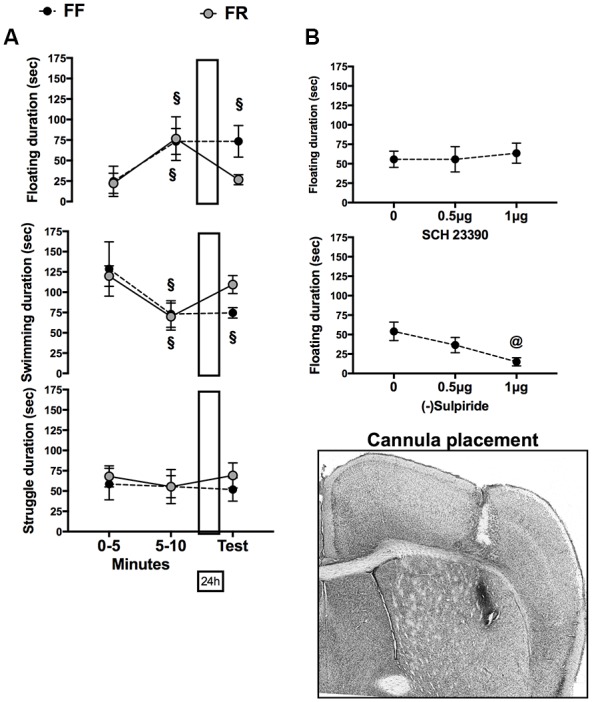
**(A)** Effects of restricted feeding on acquisition (0–5, 5–10 min) and retrieval (Test) of immobility (top), swimming (middle), and struggling (bottom) behavior. **(B)** Effects of immediate post-training infusion of the D1 receptor antagonist SCH 23390 (top) or the D2 receptor antagonist sulpiride (bottom) in the left DLS on immobility expressed on the retention test performed 24 h later. Data are expressed as mean duration (±SEM) of behavior. ^x^*p* < 0.05 vs. behavior expressed during the first 5 min of FS experience (0–5) (paired *t*-test). ^@^*p* < 0.05 vs. vehicle infusion (0) (Sidak correction for multiple comparisons).

Data on behavior expressed in FSt are presented in **Figure 3**; they indicate that whereas both FF and FR mice developed a passive coping strategy in the course of the first experience with FSt only FF mice retained this strategy for the following 24 h. Moreover, they indicate that pharmacological blockade of D2/D3R in the left DLS immediately after the first FSt experience reproduces the behavioral effects of restricted feeding in FF mice.

**Figure 3A** shows mean duration of the different behaviors expressed during the first (two blocks: 0–5, 6–10 min) and second (test: 5 min) experience of FSt by FF and FR mice. Statistical analyses revealed a significant interaction between factors (Feeding × Repeated measure) for both immobility: *F*(2,20) = 4.251; *p* < 0.05, and Swim: *F*(2,20) = 5.541; *p* < 0.05. *Post hoc* comparisons (paired *t*-test) revealed a significant increase of immobility and a significant reduction of swimming between the first and second 5 min of the first FSt experience regardless of the feeding condition. Levels of immobility and swimming were still significantly different from those expressed during the first 5 min of FSt experience in FF mice tested 24 h later but not in FR mice (**Figure 3A**).

In **Figure 3B** are reported data on the effects of immediately post-FSt unilateral infusion of SCH23390 (D1 antagonist) or Sulpiride (D2/D3 antagonist) in the left DLS on immobility expressed on the retrieval test performed 24 h later. Statistical analyses did not reveal any significant effect of the D1R antagonist whereas infusion of the D2/D3 antagonist dose-dependently reduced the amount of immobility expressed on the retrieval test [*F*(2,17) = 4.45; *p* < 0.05].

## Discussion

Major findings of the present study are: (1) the decrease of D2R mRNA in the left DLS of FR mice; (2) the selective inhibition of FSt-induced c-fos expression in the left DLS of FR mice; (3) the shared ability of pharmacological blockade of D2/D3R in the left DLS and of previous experience of reduced food availability to prevent 24 h retention of FSt-induced immobility.

In a first set of experiments we observed a selective reduction of D2R mRNA levels in the left DLS of FR mice. To the best of our knowledge this is the first report of a reduction of striatal D2R availability following exposure to adverse environmental conditions in mice. Indeed, previous studies using different chronic/repeated stress protocols and different measures of receptors availability reported an increase of D2R in the ventral striatum and no significant changes in the dorsal striatum of stressed mice (Cabib et al., 1988, 1998; Lim et al., 2011; Patrono et al., 2015).

Reduced striatal D2R availability is considered a main marker of addiction-associated aberrant plasticity because it has been reported in human addicts (Martinez et al., 2004; Tomasi and Volkow, 2013), in monkeys after chronic escalating methamphetamine (Groman et al., 2012) or prolonged cocaine self-administration (Nader et al., 2006), and in rats following cocaine self-administration (Besson et al., 2013). Thus, the finding of the present study is coherent with the ability of restricted feeding to produce phenotypes fostered by prolonged exposure to addictive drugs in laboratory animals (Carr, 2011; Zheng et al., 2012; Branch et al., 2013). Indeed, mice exposed to the protocol used for the present experiments show behavioral markers of addiction-associated aberrant neuroplasticity (Cabib et al., 2000).

Because of previous observations that the splicing of the D2R gene might influence addiction liability in humans (Moyer et al., 2011) and is influenced by exposure to addictive drugs in mice (Giordano et al., 2006), we measured the relative abundance of the two D2R isoforms. Our finding that both D2L and D2S undergo a similar decrease of their expression level in the left DLS of FR mice do not support an effect of restricted feeding on D2R gene splicing, pinpointing a possible modulation of the transcriptional activation of this gene.

Finally, the present study revealed that FR-induced decrease of D2R mRNA was confined to the left DLS. This finding could explain previous failure to demonstrate significant effects of stress on DLS D2R availability (Cabib et al., 1988, 1998; Lim et al., 2011; Patrono et al., 2015). Moreover, it is in line with evidences of lateralized brain stress responses (Berridge et al., 1999; Sullivan and Dufresne, 2006; Cerqueira et al., 2008; Lupinsky et al., 2010) and with lateralization of mesostriatal DA transmission (Schneider et al., 1982; Molochnikov and Cohen, 2014). To this regard, it is worth pointing out that higher levels of D2R have been observed in the left striatum in both healthy humans and rats (Schneider et al., 1982; Tomer et al., 2008), a phenomenon confirmed by results of the present experiments in the DLS of FF but not FR mice.

Because of our previous finding of a main role of the left DLS in consolidation of immobility acquired in FSt (Colelli et al., 2014), the selective reduction of D2R in the left DLS of FR mice was strongly suggestive of an alteration of the memory processes engaged by FSt in these mice. Therefore, we evaluated the effects of a previous FR experience on development and 24 h retention of FSt-induced passive coping. The results obtained indicated a selective effect of restricted feeding on retention rather then on expression or acquisition of the coping strategy (**Figure 3A**), supporting a role for altered memory processing in the impaired coping with FSt observed in FR mice.

The experience of restricted feeding also abolished FSt-induced c-fos expression in the left DLS (**Figure 2**). Indeed, in line with previous findings (Colelli et al., 2014), FF mice responded to a first 10 min-long FSt experience with increased c-fos expression in the left DLS a response that was not observable in FR mice. The effect of restricted feeding on c-fos expression induced by a first experience of forced swim support the hypothesis of altered consolidation of a newly acquired passive coping strategy. Indeed, expression of c-fos has been associated and causally related with consolidation of long-term memories (Lamprecht and Dudai, 1996; Bilang-Bleuel et al., 2002; Guzowski, 2002; He et al., 2002; Herry and Mons, 2004; Katche et al., 2010; Stafford et al., 2012; Reul, 2014; Saunderson et al., 2016). On the other hand, there is strong evidence for a major involvement of D2R in DA-dependent induction of striatal c-fos expression (Badiani et al., 1999; Bertran-Gonzalez et al., 2008; Kharkwal et al., 2016), thus these finding indirectly support a role for reduced D2R availability and reduced FSt-induced c-fos-expression in the left DLS of FR mice.

In a final set of experiments we directly tested the hypothesis that reduced D2R availability in the left DLS impair consolidation of passive coping acquired in FSt. To this aim we infused either a D2/D3 or of a D1 receptors antagonist in the left DLS immediately after the first FSt experience because post-training manipulations influence the consolidation of learning as long-term memory without affecting retrieval (McGaugh, 2000). The results obtained demonstrated that selective blockade of D2/D3R in the left DLS prevents 24 h retention of the immobility response acquired in the first FSt experience by FF mice. Indeed unilateral infusion of sulpiride, but not of SCH23390, in the left DLS dose-dependently reduced immobility expressed on the retention test performed 24 h later.

It should be pointed out that although sulpiride is a mixed D2/D3 receptors antagonist, there are very few D3R in the DLS (Murray et al., 1994; Erritzoe et al., 2014). Moreover, stimulation of D3R has been shown to interfere with all types of learning whereas stimulation of D2R facilitates consolidation of lasting memory (Sigala et al., 1997; Manago et al., 2009; Nakajima et al., 2013; Vicente et al., 2016). Therefore, the findings of this set of experiments offer support to a causal relationship between reduced availability of D2R in the left DLS of FR mice and impaired consolidation of coping acquired in FSt.

## Conclusion

The results of the present study support the conclusion that reduced dorsal striatal D2R availability fostered by a proximal adverse experience in adult mice can disrupt consolidation of newly acquired coping strategies leading to expression of dysfunctional coping on subsequent encounters with the stressor.

This conclusion has high translational value. Indeed, unseasonal reduction of food availability represents an unpredictable stressor in natural settings (Wingfield and Kitaysky, 2002), thus it models an ecologically meaningful stressor in laboratory settings. Moreover, altered learning mechanisms have been involved in the development of different behavioral disturbances (Robbins and Everitt, 2002; Goodman et al., 2014; Gillan et al., 2016). In addition, low striatal D2R availability has been reported in behavioral disturbances not associated with experience of addictive drugs (Wang et al., 2001; Denys et al., 2004; Schneier et al., 2007; Perani et al., 2008; Tomasi and Volkow, 2013; Broft et al., 2015). Finally, there is strong evidence that the brain circuit supporting consolidation of passive coping in FSt is also involved in response extinction (Campus et al., 2015, 2016; Goodman et al., 2016), suggesting a general role of reduced dorsal striatal D2R in liability to relapse into maladaptive behavior.

## Author Contributions

PC and SiC designed the research and analyzed and interpreted the data; PC and CO performed behavioral experiments; PC performed c-fos experiments as well as pharmacological experiments, MF and SoC collected, analyzed and interpreted data on striatal levels of D2R mRNA; SP-A, CO, and MF revised the work critically for important intellectual content. SiC wrote the manuscript.

## Conflict of Interest Statement

The authors declare that the research was conducted in the absence of any commercial or financial relationships that could be construed as a potential conflict of interest.

## Footnotes

1 http://www.mbl.org/atlas165/atlas165_start.html
